# Pore structure and fractal characteristics of deep shale gas reservoirs in the Western Chongqing block, Sichuan Basin

**DOI:** 10.1371/journal.pone.0342662

**Published:** 2026-02-12

**Authors:** Feifei Fang, Sijie He, Jie Zhang, Xizhe Li, Hua Zhang, Yan Chang, Xin Li

**Affiliations:** 1 School of Petroleum Engineering, Chongqing University of Science and Technology, Chongqing, China; 2 School of Energy Resources, China University of Geosciences (Beijing), Beijing, China; 3 Research Institute of Petroleum Exploration and Development, PetroChina, Beijing, China; 4 Institute of Porous Flow and Fluid Mechanics, University of Chinese Academy of Sciences, Langfang, China; 5 Research Institute of Jilin Oilfield Company, Songyuan, China; Northeastern University, CHINA

## Abstract

To date, researchers have not systematically investigated the geological characteristics of deep shale gas reservoirs in the Western Chongqing Block. Furthermore, the single technique remains insufficient for characterizing the complexity of their multi-scale pore structures. Therefore, this study integrates scanning electron microscopy (SEM), argon ion polishing-field emission scanning electron microscopy (AIP-FESEM), high-pressure mercury intrusion (HPMI), and low-pressure gas adsorption (LPGA) to qualitatively and quantitatively investigate the microscopic pore structure of shale gas reservoirs in the Western Chongqing block. Meanwhile, the pore fractal characteristics were analyzed based on HPMI and LPGA experiments using the mercury saturation model, the Frenkel-Halsey-Hill (FHH) model, and the volume-surface area (V-S) model. The results show that, first, the pore types of the samples in the Western Chongqing block include organic pores, intergranular pores, intragranular pores, intercrystalline pores, and interlayer fractures; second, micropores are the main contributors to the total pore volume, mainly developed in the three ranges of 0.45 ~ 0.5 nm, 0.55 ~ 0.6 nm, and 0.8 ~ 0.85 nm, followed by mesopores and finally macropores; finally, the macropores of the samples exhibit stronger heterogeneity and more complex pore-throat structures compared to mesopores. The heterogeneity of the pore structure is stronger than that of the pore surface, indicating a more complex internal pore structure. Additionally, the microporous structures of the samples are also characterized by relatively complex. The experimental results provide important guidance for the economical and efficient development of shale gas.

## 1. Introduction

China possesses abundant shale gas resources with significant potential for exploration and development [1]. It represents a critical breakthrough for advancing the unconventional energy revolution and serves as an important strategic direction for ensuring national energy security [2]. Accelerating the exploration and development of shale gas is a strategic measure to implement the guiding spirit, such as “vigorously advancing oil and gas exploration and development” and “the rice bowl of energy must be in our own hands [3].”The shale gas in the Wufeng-Longmaxi formations, with a burial depth of less than 3500 m in the south Sichuan area, has achieved economies of scale development. In particular, the Changning, Weiyuan, and Fuling blocks aim to maintain long-term stable production through three-dimensional development, repeated fracturing, and rolling-edge extension [4]. The geological resources of shale gas in China are 123.01 × 10^12^ m^3^, with the geological resources of deep shale gas totaling 55.45 × 10^12^ m^3^. The Sichuan Basin and its surrounding areas account for more than 60% of this total [5]. The evaluation and increase of production for deep shale gas in the Zigong, Luzhou, Yibin, and Dazu blocks are being actively promoted, positioning them as key strategic areas for China’s increase in shale gas reserves and production [6].

Currently, the methods commonly used to characterize the microstructure of shale include HPMI, LPGA, nuclear magnetic resonance (NMR), micro/nano computed tomography (CT) scanning, FESEM, and small-angle neutron scattering (SANS) [7–10]. Among these, HPMI and LPGA are fluid intrusion methods, FESEM is an image analysis method, while NMR, micro/nano CT scanning, and SANS are non-interference methods. Compared with conventional reservoirs, shale gas reservoirs usually develop nano-to-micron multi-scale pores and micro-fractures. The microscopic pore structure is complex, with strong heterogeneity, and the research scale of a single technique is limited [11,12]. Some scholars have used qualitative-quantitative methods to study the pore structure of shale comprehensively. Zhang et al. [13] did not simply connect the HPMI and LPGA data by dividing the connection points but applied weighted average processing on the overlapping intervals of each technique. In addition, combined with FESEM and nano-CT scanning, the pore morphology and connectivity of Nenjiang shale were directly analyzed. Liu et al. [14] qualitatively and semi-quantitatively described the morphological characteristics of shale by FESEM. Additionally, combined with the dominant pore size range of HPMI and low-pressure N_2_ adsorption (LPN_2_A) test, the pore size distribution was quantitatively measured at full scale. He et al. [15] redefined the pore size classification of shale, proposing that pores smaller than 10 nm are considered micropores, those between 10 and 50 nm are mesopores and pores larger than 50 nm are macropores. Under this standard, the development characteristics of shale micro-nano pores, including pore type, size, and shape, were directly observed through FESEM. Subsequently, by combining HPMI and low-pressure N_2_ adsorption (LPN_2_A) data, the NMR *T*_2_ spectrum was calibrated to achieve a full-scale quantitative characterization of the pores. Zhao et al. [16] conducted a comprehensive investigation on the pore characteristics of typical lacustrine shales in the northern Songliao Basin using high-pressure mercury intrusion, N₂ adsorption, focused ion beam–scanning electron microscopy (FIB-SEM), and nuclear magnetic resonance (NMR). The study focused on pore types, porosity, pore volume, specific surface area, and pore size distribution. Zhu et al. [17] used SEM, mercury intrusion porosimetry, LPGA, and logging data to conduct multi-scale quantitative characterization of OM-hosted pores. Li et al. [18] systematically studied the fractal characteristics and pore structure characteristics of Longmaxi Formation shale in southern Sichuan, including pore type, pore size distribution, and pore structure parameters, using SEM, LPGA, and NMR. Wang et al. [19] observed the sedimentary structure, pore type, and micro-fracture of Bossier shale using the petrographic microscope and SEM. Then, the pore structure parameters, such as pore size distribution, porosity, and specific surface area, were measured by mercury intrusion, SANS, and LPGA.

Currently, there has been no systematic evaluation of the structural, stratigraphic, and reservoir characteristics of the deep shale gas reservoirs in the Western Chongqing block. Meanwhile, single characterization methods are insufficient to reveal the multi-scale microscopic pore structure of the reservoirs fully. Therefore, this paper comprehensively uses SEM, AIP-FESEM, HPMI, and LPGA to study the microscopic pore structure of shale gas reservoirs in western Chongqing from both qualitative and quantitative aspects. Additionally, based on the difference in pore structure at different scales, targeted fractal models are selected to determine the fractal characteristics of shale pores at various scales. The research results provide a theoretical basis for the efficient development of shale gas.

## 2. Regional geological overview

### 2.1. Structural characteristics

**Fig 1:**
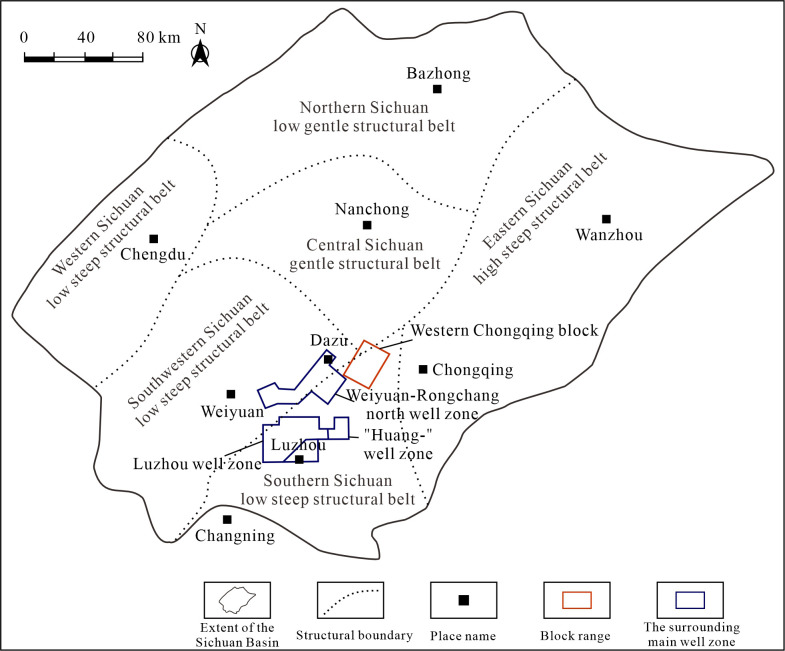
Geographical location diagram of well zone in the Western Chongqing block (Modified from “Map of China”, Map Review Number: GS(2023)2767 and “Map of China’s Topography”, Map Review Number: GS(2016)1609; Source: China Standard Map Service - http://bzdt.ch.mnr.gov.cn/).

The Sichuan Basin is a large multi-cycle superimposed basin developed on the Upper Yangtze Craton, with a total area of approximately 19 × 10^4^ km^2^. From west to east, the Sichuan Basin can be divided into six structural units: the Western Sichuan low steep structural belt, the Southwestern Sichuan low steep structural belt, the Central Sichuan gentle structural belt, the Northern Sichuan low gentle structural belt, the Southern Sichuan low steep structural belt, and the Eastern Sichuan high steep structural belt [20]. As shown in Fig 1, the Western Chongqing block is located southeast of the Central Sichuan gentle structural belt and northwest of the Southern Sichuan low steep structural belt [21]. The structure is distributed in a band on the plane, and the whole shows the structural characteristics of the NE-SW trending graben and horst alternately. The buried depth of Wufeng-Longmaxi shale reservoirs in this block is usually between 3500 m and 4500 m, which is a typical deep shale gas reservoir [22].

### 2.2. Stratigraphic characteristics

The basal boundary of the Longmaxi Formation in the Sichuan Basin is conformably in contact with the Upper Ordovician Wufeng Formation, with the lithological boundary marked by the shell limestone of the Guanyinqiao Member at the top of the Wufeng Formation. Due to the different degrees of denude of Silurian strata in the Western Chongqing area, the Longmaxi Formation is in contact with different overlying strata [23]. For example, in the Zu201 well zone, the Longmaxi Formation is in unconformable contact with the overlying Permian Liangshan Formation, while the Zu203 well zone is in conformable contact with the overlying Silurian Shiniulan Formation. According to the sedimentary cycle, the Longmaxi Formation can be divided from bottom to top into Long1 and Long2 members. The boundary is the grey-black shale at the bottom of the Long2 member and the rhythmic layer of black shale-grey silty shale interbeds in the Long1 member. The Long1 member is a progradational reverse cycle formed by continuous regression. According to the sedimentary cycle and lithological characteristics, it can be divided from bottom to top into the Long1_1_ and Long1_2_ sub-members [24]. In which, the Long1_1_ sub-members can be further subdivided into four sub-layers: Long1_1_^1^, Long1_1_^2^, Long1_1_^3^ and Long1_1_^4^ from bottom to top.

### 2.3. Reservoir characteristics

The average fragile mineral content in the Wufeng Formation–Long1_1_ sub-member reservoirs in each of the well zones of the western Chongqing block ranges from 65% to 72%, indicating a high-fragile shale with favorable fracability. The total organic carbon (TOC) content averages between 2.7% and 3.5%, classifying it as a high-TOC shale with strong adsorption capacity. The average porosity ranges from 4.3% to 5.2%, placing it in the category of medium to low porosity shales, which suggests relatively limited reservoir capacity. The average gas saturation exceeds 60%, reflecting favorable gas-bearing properties. The average gas content ranges from 4.4 to 5.6 m^3^/t, indicating a high production potential. The key geological parameters of the Wufeng Formation-Long1_1_ sub-member in each well zone of the Western Chongqing block are shown in Table 1.

**Table 1:** Reservoir characteristic parameters of the Wufeng Formation-Long1_1_ sub-member in each well zone of the Western Chongqing block.

Well zone	Fragile mineral content/%	TOC/%	Porosity/%	Gas saturation/%	Gas content/(m^3^/t)
Zu210	65 ~ 70 (67)	2.7 ~ 2.8 (2.8)	4.0 ~ 4.9 (4.7)	60 ~ 65 (63)	4.5 ~ 5.5 (5.0)
Zu208	65 ~ 70 (68)	2.8 ~ 2.9 (2.9)	4.2 ~ 6.4 (5.2)	62 ~ 67 (66)	5.0 ~ 6.5 (5.6)
Zu206	70 ~ 78 (72)	2.7 ~ 2.9 (2.8)	4.2 ~ 5.5 (5.0)	62 ~ 67 (64)	4.4 ~ 6.5 (4.9)
Zu203	64 ~ 73 (69)	2.7 ~ 2.9 (2.8)	4.0 ~ 4.7 (4.4)	60 ~ 66 (63)	4.2 ~ 4.6 (4.4)
DA3	65 ~ 78 (70)	1.0 ~ 8.2 (3.5)	1.5 ~ 7.5 (4.3)	62 ~ 71 (66)	1.9 ~ 11.9 (5.3)
Huang202	59 ~ 75 (65)	2.5 ~ 3.2 (2.7)	4.1 ~ 5.2 (4.7)	60 ~ 69 (63)	5.0 ~ 6.0 (5.4)

The mineral composition of the shale reservoirs in the Wufeng Formation-Long1_1_ sub-member in the Western Chongqing block is dominated by silicoide (quartz and feldspar), carbonate minerals (calcite and dolomite), and clay minerals, with minor amounts of pyrite [24]. The clay minerals mainly include illite and chlorite. The free gas of shale reservoirs with a buried depth of 3500 ~ 4000 m in the Western Chongqing block accounts for 61%, and the free gas of shale reservoirs with a buried depth of 4000 ~ 4500 m accounts for 57%, indicating that the shale gas in the Western Chongqing block is mainly free state [25].

## 3. Materials and methods

### 3.1. Materials

Two shale samples were collected from the Long1_1_ sub-member in a well zone of the Western Chongqing block. The samples were analyzed using SEM, AIP-FESEM, LPGA, and HPMI techniques.

### 3.2. Methods

The SEM experiment was conducted by thermo scientific scanning electron microscope to observe the structure, crystal form and mineral type of the sample. The FESEM experiment was conducted using the Nova NanoSEM 450 field emission scanning electron microscope to analyze the pore type. The LPGA experiment was conducted by the ASAP2460 adsorption instrument, while the HPMI experiment was conducted by the AutoPore IV 9510 automatic mercury intrusion instrument. The specific operation process of the above experiments has been fully discussed in the existing research, so this article will not repeat. The relevant content can be found in detail in the reference [26–28].

International Union of Pure and Applied Chemistry (IUPAC) classifies nanopores into three categories: micropores with pore sizes <2 nm, mesopores with pore sizes between 2 and 50 nm, and macropores with pore sizes >50 nm [29]. Studies have shown that the pore size range characterized by HPMI is between 3.6 nm and 950 μm, LPN_2_A is between 2 nm and 200 nm, and low-pressure CO_2_ adsorption (LPCO_2_A) is between 0.35 nm and 2.00 nm [30]. Therefore, HPMI is usually used to characterize the macropores of shale, LPN_2_A is used to characterize the mesopores of shale, and LPCO_2_A is used to characterize the micropores of shale.

### 3.3. Fractal theory

The fractal theory was first proposed by Mandelbrot [31], which is now widely used to describe complex geometry with self-similarity. Literature studies have shown that natural porous media also have fractal characteristics in a specific scale range [32,33]. Fractal theory can measure the irregularity of complex geological bodies at the micro-scale, characterize the order and complexity of the microscopic morphology of geological bodies. The fractal dimension of porous media typically falls between 2 and 3. A value closer to 2 indicates that the pore structure is relatively simple, with smoother surfaces, fewer fine structures, and a smaller spatial distribution range. Conversely, a value approaching 3 suggests a more complex pore structure, rougher surfaces, more fine structures, and a wider spatial distribution range [34,35]. The fractal dimension can be derived from various data sources and models; however, it is essential to select appropriate fractal models based on the specific pore structure characterization method and scale to determine the fractal characteristics of shale pores accurately.

The fractal dimension of HPMI experimental data is calculated by the mercury saturation method. The formula is [36]:


$$\mathrm{lg}\left(1-S_{Hg}\right)=(D_{M}-3)\mathrm{lg}P_{c}-(D_{M}-3)P_{\min}$$ (1)

In which: *S*_Hg_-Mercury saturation, %; *D*_M_-Mercury fractal dimension; *P*_c_-capillary pressure, MPa; *P*_min_-Capillary pressure corresponding to the maximum pore throat radius rmax, MPa.

The fractal dimension of N_2_ adsorption experimental data is calculated by the FHH model. The formula is [37]:


$$\ln\;V=\left(D_{N}-3\right)\;\cdot \;\ln\left(\ln\frac{P_{0}}{P}\right)\;\;+\;C$$ (2)

In which: *V*-Gas adsorption capacity, cm^3^/g; *D*_N_-FHH fractal dimension; *P*_0_- Gas saturated vapor pressure, MPa; *P*-System equilibrium pressure, MPa; C-Constant.

The fractal dimension of CO_2_ adsorption experimental data is calculated by the V-S model. The formula is [38]:


$$\ln\;V=\frac{\text{3}}{D_{C}}\;\cdot \;\ln\;\;S+C$$ (3)

In which: *V*-Gas adsorption capacity, cm^3^/g; *D*_C_-V-S fractal dimension; *S*-Cumulative specific surface area, m^2^/g; C-Constant.

## 4. Study on microscopic pore structure characteristics of deep shale gas in Western Chongqing block

### 4.1. SEM-based characterization of microscopic pore structures

**Fig 2:**
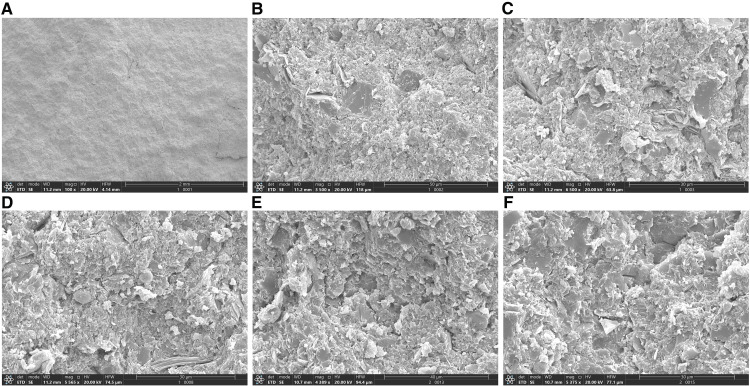
SEM images of deep shale in the Western Chongqing block. **(a)** X100 **(b)** X3500 **(c)** X6500 **(d)** X5565 **(e)** X4389 **(f)** X5375.

In the overall view, the sample structure is dense, with siliceous aggregates displaying a mud-crystalline structure (Fig 2a). Upon magnification, the siliceous aggregate displays a mud-crystalline structure entrainment with quartz particles (Fig 2b), siliceous crystals are tightly mosaicking contact (Fig 2c), entrainment with spherical granular pyrite crystal aggregates (Fig 2d), individual micro-fractures (Fig 2e), and micro-pore fractures (Fig 2f).

### 4.2. AIP-FESEM-based characterization of microscopic pore structures

**Fig 3:**
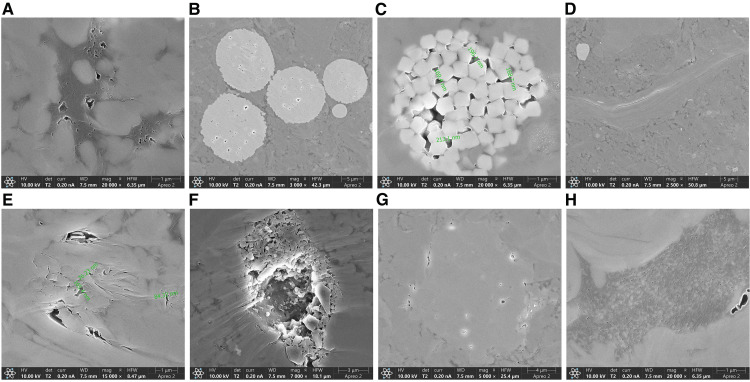
AIP-FESEM images of deep shale from the Western Chongqing block. **(a)** X20000 **(b)** X3000 **(c)** X20000 **(d)** X2500 **(e)** X15000 **(f)** X7000 **(g)** X5000 **(h)** X20000.

As shown in Fig 3a, the black areas represent organic matter, within which organic pores are developed, and intergranular pores between organic and inorganic particles at the black and grey junction regions. In addition, intragranular pores of pyrite particles (Fig 3b), intercrystal pores of pyrite particles (Fig 3c), interlayer fractures of clay minerals(Fig 3d), micropores and microfractures between clay mineral particles (Fig 3e), dense intergranular pores and fractures mixed by clay minerals at the edge of dissolution pores(Fig 3f), intergranular pores and fractures at the edge of carbonate mineral particles(Fig 3g), and dense intergranular pores generated by shallow organic matter and inorganic minerals(Fig 3h) were also developed.

### 4.3. HPMI-based characterization of microscopic pore structures

#### 4.3.1. Qualitative analysis of capillary pressure curves.

**Fig 4:**
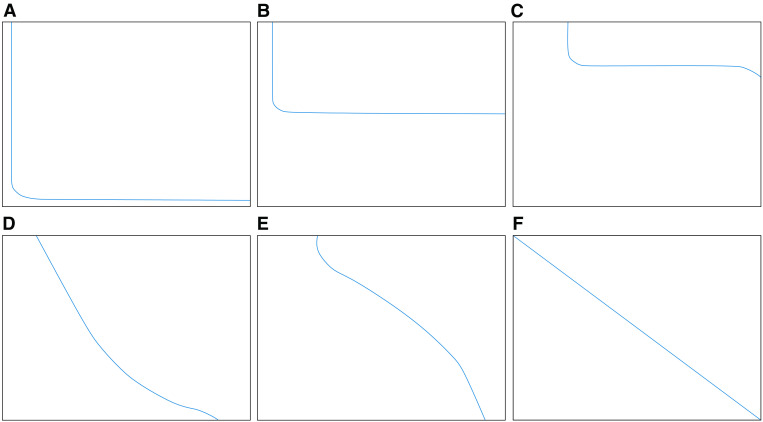
Simplify the capillary pressure curve chart [39]. **(a)** Well-sorted and coarse skewness **(b)** Well-sorted **(c)** Well-sorted and fine skewness **(d)** Poorly sorted and slight coarse skewness **(e)** Poorly sorted and slight fine skewness **(f)** Unsorted.

The qualitative characteristics of the capillary pressure curve can generally be divided into the initial section, the middle gentle section and the end upward section, and its shape is comprehensively determined by the sorting of the pore throat and the size of the throat. As shown in Fig 4, the longer and more parallel the middle gentle section of the capillary pressure curve is to the horizontal axis, the better sorting and the more concentrated pore throat size distribution. In addition, the closer the overall curve is to the lower left, the more it exhibits coarse skewness, indicating a larger pore throat radius. In contrast, the closer it is to the upper right, the more it exhibits fine skewness, indicating a smaller pore throat radius.

**Fig 5:**
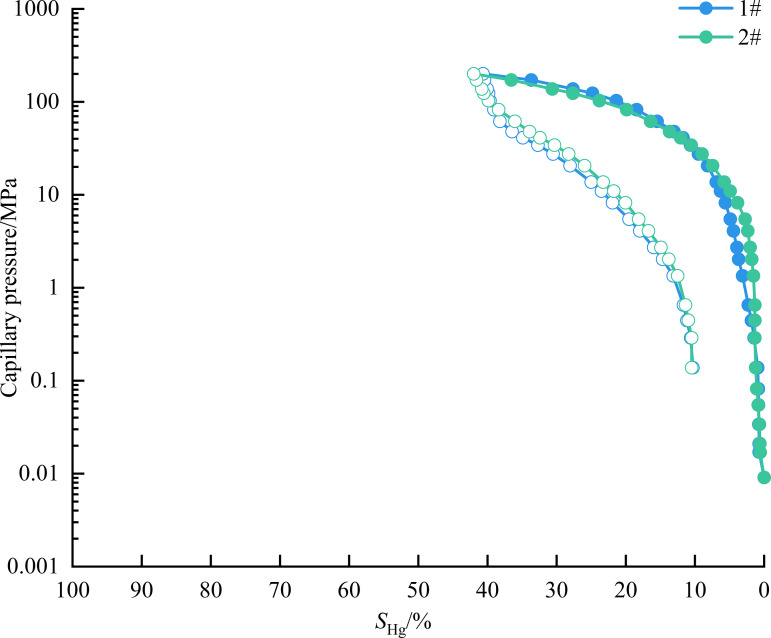
Capillary pressure curve of experimental sample.

As shown in Fig 5, the capillary pressure curves of the two samples are generally similar in shape, featuring only the initial section and the middle gentle section, without the end upward section. Referring to the simplified capillary pressure curve chart (Fig 4), the mercury injection curve of the sample was qualitatively analyzed, revealing that both samples exhibited moderate sorting with a slight fine skewness. In the low-pressure section (0 ~ 15 MPa), the increase in mercury saturation is slow, indicating that the macropore development degree is low in this range. In the medium- and high-pressure section (10 ~ 200 MPa), mercury saturation increased rapidly, indicating that the mesopore development degree is high in this range. In addition, the mercury withdrawal curve is the type II pore in the research results of Chen et al. [40], which belongs to the relatively poor shale gas reservoir section and is more conducive to desorption, diffusion, and seepage.

#### 4.3.2. Capillary pressure curves characteristic parameters.

The capillary pressure curve contains several characteristic parameters, which scholars generally categorize into three categories: pore throat size, pore throat distribution, and pore throat connectivity [41]. The pore throat size characteristic parameters include the threshold pressure and the maximum pore throat radius; the pore throat distribution characteristic parameters include sorting coefficient and skewness; the pore throat connectivity characteristic parameters include maximum mercury saturation and mercury withdrawal efficiency. The definition and significance of the characteristic parameters are detailed in Table 2.

**Table 2:** Classification, definitions, and significance of capillary pressure curves characteristic parameters.

Classification	Characteristic parameters	Definitions	Significance
Pore throat size	Threshold pressure	The minimum pressure when mercury enters into the core	Determine the development type of shale nanopores
	Maximum pore throat radius	The throat radius corresponding to the threshold pressure	
Pore throat distribution	Sorting coefficient	Concentration degree of pore throat geometry size	The smaller the value, the more concentrated the geometric size of the pore throats
	Skewness	Measure of the asymmetric degree of pore throat size distribution	A positive value indicates coarse skewness, a negative value indicates fine skewness, and 0 indicates a symmetrical distribution
Pore throat connectivity	Maximum mercury saturation	The mercury saturation value at the maximum applied pressure	Reflect the sample storage capacity
	Mercury withdrawal efficiency	The ratio of mercury withdrawal volume to mercury intrusion volume	Reflect sample connectivity

As shown in Table 3, the 1# sample exhibits poor physical properties, with porosity and permeability of 1.29% and 0.0069 mD, respectively. The threshold pressure and the maximum pore throat radius are 13.75 MPa and 0.053 μm, respectively, indicating that the sample develops mesopores and macropores. The sorting coefficient and skewness were 2.893 and −0.736, respectively, indicating that the sample had poor sorting and slight fine skewness. The maximum mercury saturation and mercury withdrawal efficiency were 40.66% and 74.67%, respectively, indicating that the sample storage capacity was relatively poor, but the connectivity was good. The physical properties of the 2# sample are slightly better than those of 1#, and the porosity and permeability are 1.32% and 0.0101 mD, respectively. The threshold pressure and the maximum pore throat radius are 5.48 MPa and 0.134 μm, respectively, indicating a higher degree of mesopore and macropore development in the 2# sample compared to the 1# sample. The sorting coefficient and skewness were 1.984 and −0.605, respectively, indicating that the sample had good sorting and slightly fine skewness. The maximum mercury saturation and mercury withdrawal efficiency were 41.99% and 75.02%, respectively, indicating that the sample storage capacity and connectivity were slightly better than the 1# sample.

**Table 3:** Capillary pressure curve characteristic parameters.

Numbering	Porosity/%	Permeability/mD	Threshold pressure/MPa	Maximum pore throat radius/μm	Sorting coefficient	Skewness	Maximum mercury saturation/%	Mercury withdrawal efficiency/%
1#	1.29	0.0069	13.75	0.053	2.893	−0.736	40.66	74.67
2#	1.32	0.0101	5.48	0.134	1.984	−0.605	41.99	75.02

#### 4.3.3. Pore size distribution of the HPMI method.

Referring to the Chinese national standards GB/T 29171−2023 “Determination of Rock Capillary Pressure Curve” [42] and GB/T 21650.2−2008 “Determination of Pore Size Distribution and Porosity of Solid Materials by Mercury Intrusion and Gas Adsorption Methods – Part 1: Mercury Intrusion Method” [43], the capillary pressure curve can be converted into three forms using the Washburn equation (equation 4): the columnar frequency histogram of pore throats, the frequency distribution curve and the cumulative frequency distribution curve of the pore throat, and the volume distribution curve of pore throats [44,45].


$$P_{c}=\frac{\text{2}\sigma \text{cos}\theta }{r_{c}}=\frac{\text{0}.\text{7}\text{3}\text{5}}{r_{c}}$$ (4)

In which: *P*_c_-Capillary pressure, MPa; *σ*-Surface tension of mercury-air system, N/m; *θ*-Wetting angle between mercury and solid surface, °; *r*_c_-capillary radius, μm.

**Fig 6:**
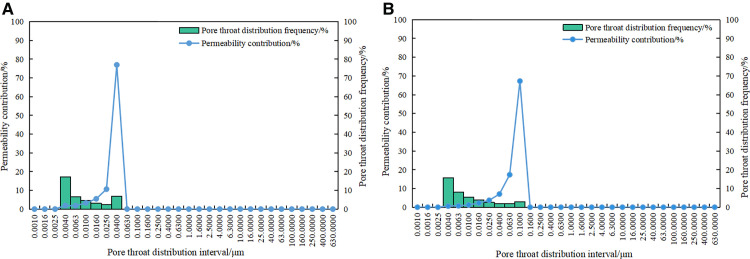
Pore-throat distribution histogram. **(a)** 1# **(b)** 2#.

As shown in Fig 6a, the pore throat radius of the 1# sample is mainly concentrated between 0.004 ~ 0.04 μm, which belongs to the definition range of mesopore and macropore. At 0.004 μm, the pore throat frequency reaches a peak of 17.09%, indicating well-developed mesopores in the sample. The permeability contribution exhibits a unimodal distribution, peaking at 76.92% at 0.04 μm. As shown in Fig 6b, the pore throat radius of the 2# sample is concentrated between 0.004 ~ 0.1 μm, and the macropore development degree is higher than that of the 1# sample. Similarly, a peak in pore throat frequency appears at 0.004 μm, reaching 15.66%, suggesting well-developed mesopores. Compared with the peak pore throat radius of permeability contribution of 1# sample, the permeability contribution value of 2# sample also shows a unimodal distribution, reaching 67.26% at 0.1 μm, indicating that permeability is mainly affected by larger pore throat.

### 4.4. LPN_2_A-based characterization of microscopic pore structures

#### 4.4.1. Qualitative description of pore structure characteristics based on LPN_2_A.

**Fig 7:**
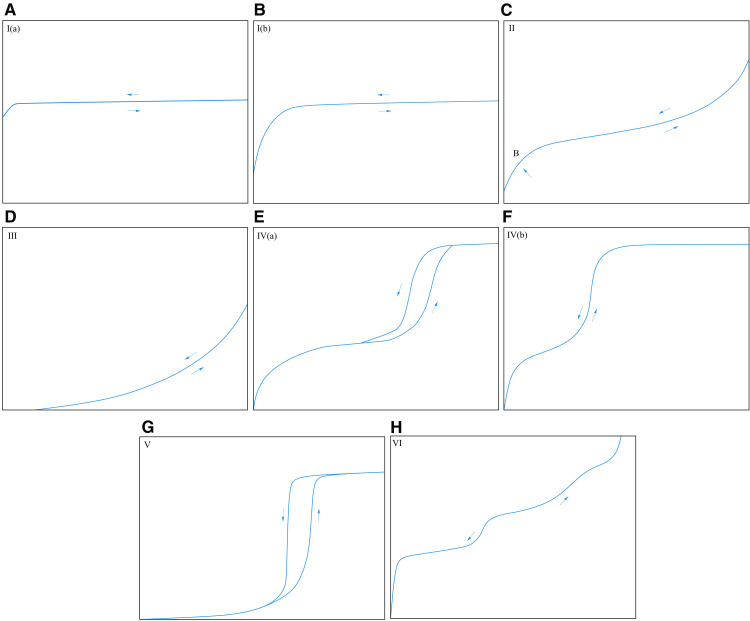
Standard adsorption isotherm classification chart [46]. **(a)** Micropore type **(b)** Micropore–narrow mesopore type **(c)** Non-pore/macropore type **(d)** Non-pore/macropore-weak interaction type **(e)** Mesopore-capillary condensation type **(f)** Narrow mesopore type **(g)** Micropore/mesopore-weak interaction type **(h)** Multilayer adsorption type.

As shown in Fig 7, the IUPAC proposed a standard adsorption isotherm classification chart [46]. Type I adsorption isotherm, also known as the Langmuir adsorption isotherm, exhibits an initial concave-down increase (micropore filling) with an increase in relative pressure, followed by nearly horizontal growth (micropore saturation) close to a limiting value. I(a) type adsorption isotherm, the sample mainly develops micropores with pore size < 1 nm. For type I(b) adsorption isotherm, the sample mainly develops micropores and narrow mesopores of < 2.5 nm. The type II adsorption isotherm, which is the “S” type, appears in non-pore or macropore samples. There is an inflection point B, indicating the end of single-layer adsorption. When the relative pressure exceeds the inflection point, it becomes multi-layer adsorption. When the relative pressure is 1, the number of adsorption layers is infinite. The type III adsorption isotherm is concave-upward on the whole, and the interaction between liquid nitrogen and the sample is relatively weak. The adsorbed molecules gather around the favorable sites on the surface of non-pore or macropore samples. When the relative pressure is 1, the adsorption capacity is a finite value. Type IV adsorption isotherm typically appears in samples with well-developed mesopores. At low relative pressures, it resembles the characteristics of a Type II isotherm, where molecules adsorb on the mesopore walls in a monolayer–multilayer manner. When the relative pressure reaches a certain value, capillary condensation occurs. Type IV (a) adsorption isotherm, when the pore size is greater than 0.4 nm, the phenomenon of adsorption hysteresis occurs, and the adsorption and desorption isotherms do not coincide, forming an “adsorption hysteresis loop” Type IV (b) mainly develops narrow mesopores, so it is reversible. Type V adsorption isotherm exhibits characteristics similar to Type III at low relative pressures, indicating relatively weak interactions between liquid nitrogen and the sample. At higher relative pressures, molecular aggregation is followed by pore filling, resulting in adsorption hysteresis similar to that observed in Type IV(a), forming an “adsorption hysteresis loop.” This type of isotherm typically appears in water adsorption on hydrophobic micropore and mesopore samples. The type VI adsorption isotherm appears in highly uniform non-pore samples, and the whole is stepped, showing layer-by-layer adsorption. The step height is the capacity of each adsorption layer, and this type of curve cannot be obtained completely by liquid nitrogen adsorption.

**Fig 8:**
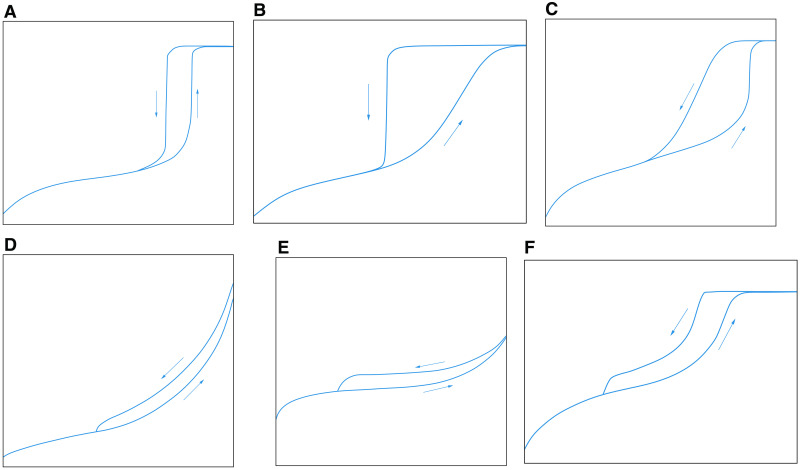
Standard adsorption hysteresis loop classification chart [46]. **(a)** H1 **(b)** H2(a) **(c)** H2(b) **(d)** H3 **(e)** H4 **(f)** H5.

As shown in Fig 8, the IUPAC proposed an adsorption hysteresis loops classification chart based on the adsorption hysteresis loops characteristics [46]. The H1-type hysteresis loop: The adsorption curve and the desorption curve are vertically nearly parallel when the relative pressure is high, and the pore morphology is cylindrical with openings at both ends. The H2-type hysteresis loop, influenced by the complex pore structure, is further subdivided into H2 (a) and H2 (b). The H2(a) hysteresis loop exhibits a pronounced convexity upward on the desorption curve, with the pore structure resembling an “ink-bottle shape” with a narrow neck. The H2(b) hysteresis loop shows a pronounced convexity down on the adsorption curve, with the pore structure resembling an “ink-bottle shape” with a wide neck. Type H3-type hysteresis loop: The adsorption curve does not exhibit a maximum adsorption amount, indicating that the pores have a strong adsorption capacity. The pore structure consists of groove pores formed by flaky particles [47]. Type H4 -type hysteresis loop: The adsorption and desorption curves extend nearly horizontally, indicating weak adsorption capacity and poor connectivity of the pores. The pore structure is characterized by slit-like pores. Type H5-type hysteresis loop: Associated with certain pore structures containing open and partially closed mesopores, the pore structure is typically characterized by irregular shapes, such as an ink-bottle shape [48].

**Fig 9:**
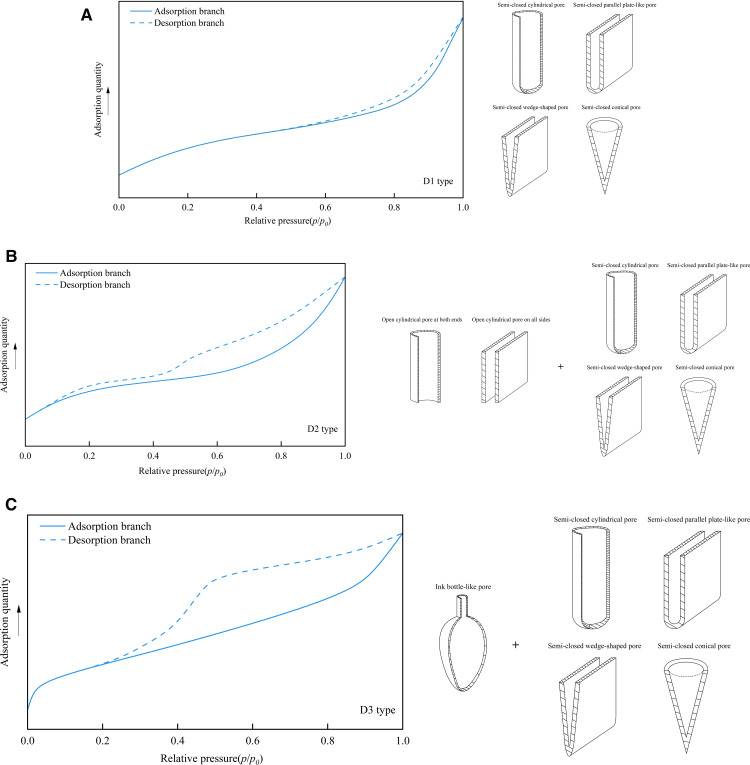
Liquid nitrogen adsorption hysteresis loop classification chart. **(a)** D1 type **(b)** D2 type **(c)** D3 type.

As shown in Fig 9, Shan et al. [49] also established a classification chart for nitrogen adsorption hysteresis loops, which was later applied by Yang and Jiao [50,51] in the analysis of shale samples. The D1-type hysteresis loop: The adsorption curve and the desorption curve basically overlap. When the relative pressure > 0.6, the adsorption and desorption curves deviate slightly. The pore morphology represented by the hysteresis loop includes semi-closed parallel plate-like pores, semi-closed cylindrical pores, semi-closed conical pores, and semi-closed wedge-shaped pores. The D2-type hysteresis loop: When the relative pressure is low, the adsorption and desorption curves basically overlap, indicating that the pores with smaller pore sizes are mainly semi-closed impermeable pores. When the relative pressure is greater than 0.5, the deviation of the adsorption and desorption curves is obvious, indicating that the pores with larger pore sizes are open pores, including two types of open cylindrical pores at both ends and open cylindrical pores on all sides, and there may be some semi-closed pores. The D3-type hysteresis loop: The pore morphology represented by this type of curve includes ink bottle pores in addition to the four semi-closed pores with the D1 hysteresis loop.

**Fig 10:**
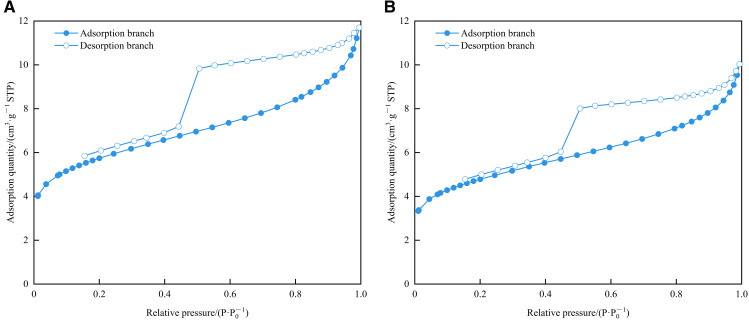
N_2_ adsorption-desorption curves of shale samples in the study area. **(a)** 1# **(b)** 2#.

As shown in Fig 10, there are three main stages of N_2_ adsorption: In the low-pressure section (0.15 < *P*/*P*_0_ < 0.45), the adsorption capacity increases linearly and slowly with the increase of relative pressure, and the adsorption and desorption curves basically overlap. The medium-pressure section (0.45 ≤ *P*/*P*_0_ < 0.9) can be subdivided into two stages. When the relative pressure is between 0.45 and 0.5, the adsorption capacity increases rapidly. When it is between 0.5 and 0.9, it is similar to that in the low-pressure section, and the adsorption capacity increases linearly and slowly. In the medium-pressure section, due to the capillary condensation phenomenon, the desorption and adsorption curves do not coincide, forming a hysteresis loop. In the high-pressure range (0.9 ≤ P/P₀ < 1), the adsorption quantity increases sharply with a concave-upward trend, and no adsorption saturation is observed even near the saturation vapor pressure. This indicates the presence of a certain amount of macropores or microfractures in the sample, which is consistent with the conclusions from HPMI. In general, the adsorption isotherm curves of 1# and 2# samples not only conform to the IV (a) isotherm and H2 (b) hysteresis loop characteristics proposed by IUPAC but also are similar to the D2 (low-pressure section) and D3 (medium-pressure section and high-pressure section) adsorption loops proposed by Shan et al. [49], indicating that the mesopores of the samples are more developed. The pores with small pore sizes (in the low-pressure section) are semi-closed impermeable pores. In contrast, the pores with large pore sizes (medium-pressure section and high-pressure section) exhibit various morphologies, including the semi-closed parallel plate-like pore, semi-closed cylindrical pore, semi-closed conical pore, and the “ink-bottle shape” with a wide neck.

#### 4.4.2. Pore size distribution of the LPN_2_A method.

**Fig 11:**
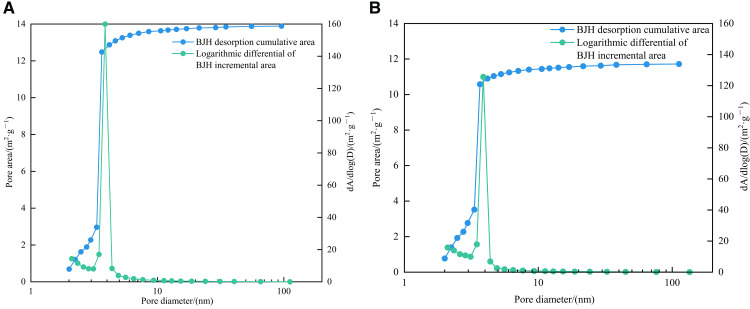
Relationship diagram between BJH desorption cumulative surface area/logarithmic differential of incremental surface area and pore diameter. **(a)** 1# **(b)** 2#.

**Fig 12:**
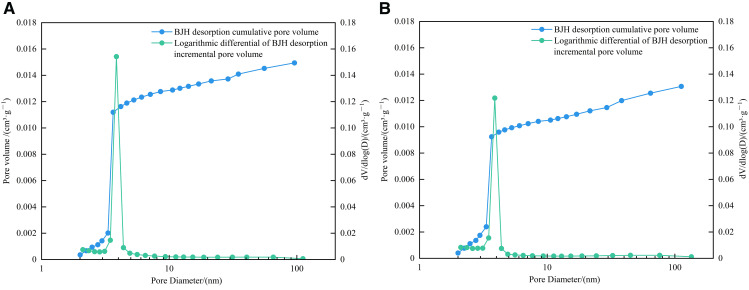
Relationship diagram between BJH desorption cumulative pore volume/logarithmic differential of incremental pore volume and pore diameter. **(a)** 1# **(b)** 2#.

The BET specific surface area of 1# and 2# samples were 18.9560 m^2^/g and 15.8663 m^2^/g, respectively. The BJH total pore volume (desorption) were 0.014948 cm^3^/g and 0.013067 cm^3^/g, respectively. The average pore diameter (desorption) was 4.3055 nm and 4.4596 nm, respectively. As shown in Figs 11 and 12, the growth rates of BJH desorption cumulative area and BJH desorption cumulative pore volume of the two samples are the most significant in the mesopore section. The most probable pore size of the 1# sample is 3.86 nm, and that of the 2# sample is 3.88 nm, indicating that the pore size of the two samples is mainly concentrated between 2 and 5 nm.

### 4.5. LPCO_2_A-based characterization of microscopic pore structures

#### 4.5.1. Qualitative description of pore structure characteristics based on LPCO_2_A.

**Fig 13:**
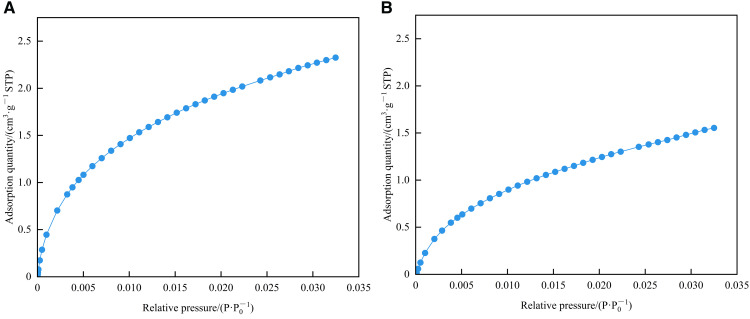
CO_2_ adsorption curves of shale samples in the study area. **(a)** 1# **(b)** 2#.

As shown in Fig 13, there are two main stages of CO_2_ adsorption: when the relative pressure is less than 0.005, the adsorption quantity of the two samples increases rapidly with the increase of relative pressure (micropore filling). When the relative pressure is greater than 0.005, the change rate of adsorption quantity gradually decreases (micropore saturation). The CO_2_ adsorption curve showed an upward convexity as a whole, which was consistent with the characteristics of the type I(b) isotherm proposed by IUPAC, indicating that the sample developed micropores and small-sized mesopores. In addition, the CO_2_ adsorbed quantity by 1# sample is higher than that of 2# sample, indicating that 1# sample contains more micropores.

#### 4.5.2. Pore size distribution of the LPCO_2_A method.

**Fig 14:**
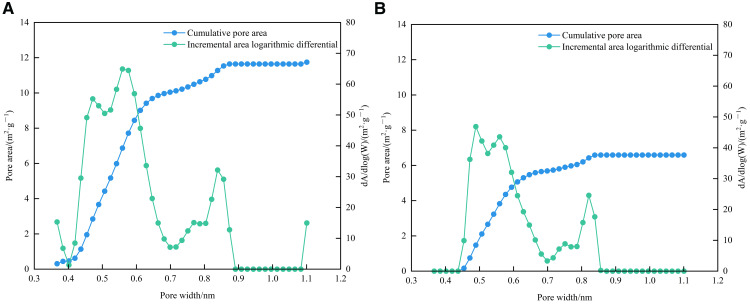
Relationship diagram between the logarithmic differential of specific surface area and pore width. **(a)** 1# **(b)** 2#.

**Fig 15:**
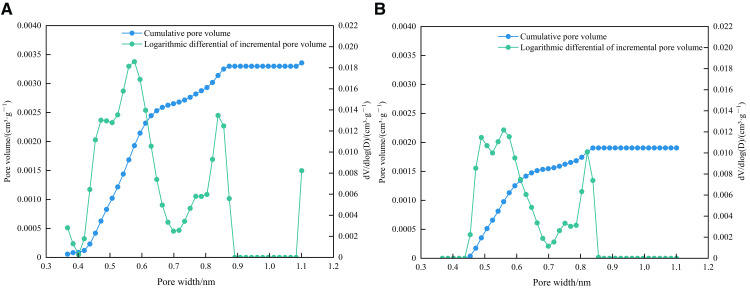
Relationship diagram between the logarithmic differential of pore volume and pore width. **(a)** 1# **(b)** 2#.

The D-R specific surface areas of 1# and 2# samples were 17.9290 m^2^/g and 14.5193 m^2^/g, respectively, and the total pore volumes were 0.00336 cm^3^/g and 0.00191 cm^3^/g, respectively. As shown in Figs 14 and 15, the development of micropores in the two samples is generally multimodal. The first main peak of the 1# sample is between 0.45 ~ 0.5 nm, and the corresponding pore size is 0.47 nm. The second main peak is between 0.55 ~ 0.6 nm, and the corresponding pore size is 0.56 nm. The third main peak is between 0.8 ~ 0.85 nm, and the corresponding pore size is 0.84 nm. The first main peak of the 2# sample is between 0.45 ~ 0.5 nm, and the corresponding pore size is 0.49 nm. The second main peak is between 0.55 ~ 0.6 nm, and the corresponding pore size is 0.56 nm. The third main peak is between 0.8 ~ 0.85 nm, and the corresponding pore size is 0.82 nm. In general, the pore volume and specific surface area of micropores are mainly provided by pores in the range of 0.45 ~ 0.5 nm, 0.55 ~ 0.6 nm, and 0.8 ~ 0.85 nm.

### 4.6. Full-scale characterization of shale pores

**Fig 16:**
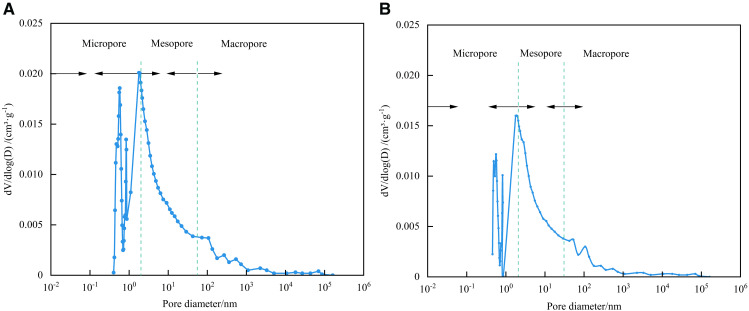
Full pore diameter distribution. **(a)** 1# **(b)** 2#.

Combined with the dominant pore size segments of HPMI, LPN_2_A, and LPCO_2_A, 2 nm and 50 nm were used as the connection points of micropore-mesopore and mesopore-macropore to characterize the full pore size of the samples. As shown in Fig 16, the full pore size distribution characteristics of the two samples are basically the same, showing multi-peak characteristics. Micropores, mesopores, and macropores contribute to the total pore volume, reflecting the complexity of shale pore structure. Micropores are the main contributors to the total pore volume, mainly developed in the three ranges of 0.45 ~ 0.5 nm, 0.55 ~ 0.6 nm, and 0.8 ~ 0.85 nm, followed by mesopores and finally macropores.

### 4.7. Fractal characteristics of shale nanopores

**Fig 17:**
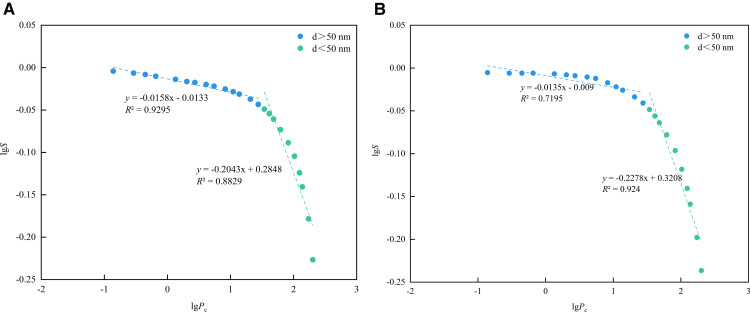
Fractal dimension fitting curve of the HPMI. **(a)** 1# **(b)** 2#.

As shown in Fig 17, the pore fractal dimension curve determined by the HPMI method exhibits an obvious two-stage pattern. Taking 50 nm as the segmentation point, the fractal dimension of the segmentation interval is calculated respectively. The fractal dimensions of macropores and mesopores are denoted as *D*_M1_ and *D*_M2_, respectively. As shown in Table 4, the average value of *D*_M1_ is 2.9854, and the average value of *D*_M2_ is 2.7840. *D*_M1_ is greater than *D*_M2_ and closer to 3, indicating that the macropores of Long1_1_ shale are more heterogeneous and the pore throat structure is more complex.

**Table 4:** Fractal dimension calculation results of the HPMI.

Number	1#	2#
Fitting curve	*y* = −0.0158*x*-0.0133	y = −0.0135x-0.009
	*y* = −0.2043*x* + 0.2848	y = −0.2278x + 0.3208
*R* ^2^	0.9295	0.7195
	0.8829	0.924
*D*_M1_	2.9842	2.9865
*D*_M2_	2.7957	2.7722

**Fig 18:**
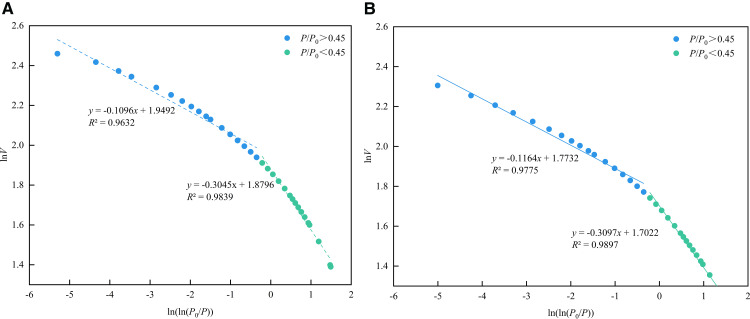
Fractal dimension fitting curve of the LPN2A. **(a)** 1# **(b)** 2#.

Combined with the range of the hysteresis loop in Fig 10, the fractal dimension in the low-pressure section (*P*/*P*₀ < 0.45) was defined as *D*_N1_, representing the surface fractal dimension, associated with monolayer adsorption dominated by van der Waals force. The high-pressure section (*P*/*P*₀ > 0.45) was defined as *D*_N2_, representing the structural fractal dimension, reflecting multilayer adsorption dominated by capillary condensation. As shown in Fig 18, the nitrogen adsorption data fitted using the FHH model yielded *R*^2^ values above 0.96, indicating a good fitting performance. As shown in Table 5, the average values of *D*_N1_ and *D*_N2_ are 2.6929 and 2.887, respectively. The higher *D*_N2_ value indicates that the heterogeneity of the pore structure in the Long1_1_ sub-member shale is greater than that of the pore surface, suggesting a more complex internal pore structure.

**Table 5:** Fractal dimension calculation results of the LPN_2_A.

Number	1#	2#
Fitting curve	*y* = −0.1096*x* + 1.9492	*y* = −0.1164*x* + 1.7732
	*y* = −0.3045*x* + 1.8796	*y* = −0.3097*x* + 1.7022
*R* ^2^	0.9632	0.9775
	0.9839	0.9897
*D*_N1_	2.6955	2.6903
*D*_N2_	2.8904	2.8836

**Fig 19:**
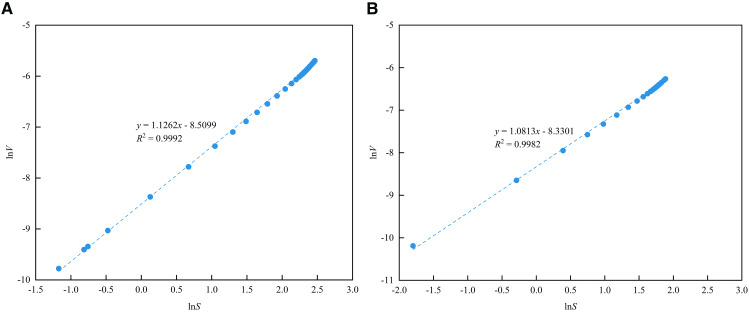
Fractal dimension fitting curve of the LPCO_2_A. **(a)** 1# **(b)** 2#.

As shown in Fig 19, the CO₂ adsorption data fitted using the V-S model yielded *R*^2^ above 0.99, indicating excellent fitting performance. As shown in Table 6, the average *D*_C_ value is 2.7191, suggesting that the micropores structure of the Long1_1_ sub-member shale is relatively complex.

**Table 6:** Fractal dimension calculation results of the LPCO_2_A.

Number	1#	2#
Fitting curve	*y* = 1.1262*x*-8.5099	*y* = 1.0813*x*-8.3301
*R* ^2^	0.9992	0.9982
*D*_C_	2.6638	2.7744

The pore structures of both shale samples follow fractal laws and exhibit self-similarity. The macropore heterogeneity is stronger than that of the mesopores and micropores (Table 7). The reason may be that the macropore has a large pore size, a wide distribution range, diverse genetic mechanisms, and is easily affected by diagenesis, so the fractal dimension is large [52].

**Table 7:** Fractal dimension calculation results summary.

Number	*D*_M1_	*D*_M2_	*D*_N1_	*D*_N2_	*D*_C_
1#	2.9842	2.7957	2.6955	2.8904	2.6638
2#	2.9865	2.7722	2.6903	2.8836	2.7744

## 5. Conclusions

By integrating multiple analytical techniques, both qualitative and quantitative analyses were conducted to investigate the micropore structure of shale gas reservoirs in the Western Chongqing Block. The results revealed that:

(1) Through SEM observation, the sample structure of the Western Chongqing block is dense, with siliceous aggregates displaying a mud-crystalline structure. Upon magnification, the siliceous aggregate displays a mud-crystalline structure entrainment with quartz particles, siliceous crystals are tightly mosaicking contact, entrainment with spherical granular pyrite crystal aggregates, individual micro-fractures, and micro-pore fractures. Through the AIP-FESEM observation, the pore types of the samples in the Western Chongqing block include organic pores, intergranular pores, intragranular pores, intercrystal pores, and interlayer fractures.
(2) Combined with the dominant pore size segments of HPMI, LPN_2_A, and LPCO_2_A, the full-scale pore size distribution of the samples was characterized. The results show that micropores are the main contributors to the total pore volume, mainly developed in the three ranges of 0.45 ~ 0.5 nm, 0.55 ~ 0.6 nm, and 0.8 ~ 0.85 nm, followed by mesopores and finally macropores.
(3) The average *D*_M1_ value (average 2.9854) is higher than *D*_M2_ (average 2.7840), and *D*_N2_ (average 2.887) is greater than *D*_N1_ (average 2.6929), while the average *D*_C_ is 2.7191. These results indicate that the macropores in the Long1_1_ sub-member shale exhibit stronger heterogeneity and more complex pore–throat structures. In addition, the overall pore structure shows greater heterogeneity than the pore surface, suggesting a more complex internal pore structure. The relatively high *D*_C_ value also reflects the complexity of the micropore structure in the shale.

The number of samples analyzed in this study is relatively limited. Subsequent experiments will continue to increase the number of samples, further study the influence factors of deep shale pore development and deep shale fractal characteristics, and conduct a comparative analysis of shale reservoir space variations at different burial depths.

## Supporting information

S1 File Supporting information. (RAR)
